# Can we really teach cognitive behavioral therapy with a massive open online course?

**DOI:** 10.1192/j.eurpsy.2020.29

**Published:** 2020-03-10

**Authors:** Thomas Gargot, Nikitas A. Arnaoutoglou, Tiago Costa, Olga Sidorova, Natasha Liu-Thwaites, Stirling Moorey, Cécile Hanon

**Affiliations:** 1 CHART Laboratory—EA 4004, THIM, Paris 8 University, Paris Lumière University, Saint Denis, France; 2 Psychiatrie de l'Enfant et de l'Adolescent, Hôpitaux Universitaires, Pitié Salpêtrière—Charles Foix, Paris, France; 3 ISIR, Sorbonne Université, Paris, France; 4 European Federation of Psychiatric Trainees Psychotherapy Working Group, Bruxelles, Belgium; 5 European Psychiatric Association Psychotherapy Section, Strasbourg, France; 6 Windsor Research Unit, Cambridgeshire, Peterborough NHS FT, Cambridge, United Kingdom; 7 Department of Psychiatry, University of Cambridge, Cambridge, United Kingdom; 8 Memory and Cognitive Functions Clinic, 1st Department of Psychiatry, "Papageorgiou" Hospital, Aristotle University of Thessaloniki, Thessaloniki, Greece; 9 Cumbria, Northumberland, Tyne and Wear, NHS Foundation Trust, Northumberland, United Kingdom; 10 Institute of Neuroscience, Newcastle University, Newcastle upon Tyne, United Kingdom; 11 Riga East University Hospital, Riga, Latvia; 12 South London and Maudsley NHS Foundation Trust, Beckenham, United Kingdom; 13 South London and Maudsley NHS Trust, Beckenham, United Kingdom; 14 Institute of Psychiatry, Psychology and Neuroscience, King’s College London, London, United Kingdom; 15 Regional Resource Center of Old Age Psychiatry West-Paris University Hospital, Corentin Celton Hospital, APHP, Paris-Descartes University, Paris, France

**Keywords:** CBT, MOOC, online, psychotherapy, training

## Abstract

A better training in psychotherapy is needed for psychiatry trainees. Online Cognitive Behavioural Therapies (CBT) could be a good solution. Free and wide audience course like Massive Open Online course (MOOCs) increase dissemination and accessibility of the training. However, the engagement needs to be improved. A hybrid approach seems relevant with the MOOC as an incentive. Beyond the promotion of the topic, a MOOC can be a promotion tool for the provider. The economic model of the MOOC needed to be taken into account to allow sustainability. To explore these elements, we take into account a survey taken during the 1st European Psychiatric Association MOOC about CBT.

## A Better Training in Psychotherapy is Needed for Psychiatry Trainees

Psychotherapy skills are fundamental for a psychiatrist. A survey of 574 psychiatry trainees, living in 22 different European countries, showed a very strong interest and motivation for psychotherapy training, but scarcity of training resources [1]. Psychotherapy training has been identified by European psychiatry trainees as their most relevant concern, coming before salary and working conditions [2].

## Online CBT Could be a Good Solution

Cognitive behavioral therapy (CBT) is a well-known and widely used manualized psychotherapy, being first-line treatment in many countries for mood disorders and other pathologies [3]. Given its widespread use and relevance, training in CBT is in great demand, including online courses [4].

Focus groups, including psychiatric trainees and young psychiatrists from Europe, have shown that online courses are seen as good opportunities to keep knowledge up-to-date. Participants value interacting with experts, as well as with their peers, believing this fosters reflective practice [5]. A survey of psychiatry trainees from 32 European countries showed that access to affordable medical information is relevant, particularly via the internet [6]. For instance, the European Federation of Psychiatric trainees is promoting online courses and summer schools [7], and provides online training material (e.g., psychotherapy guidebooks) [8]. Online courses allow flexibility in participation schedules and can be cheaper than traditional academic courses. Still, they require self-discipline and self-motivation, which might explain the high dropout rates [9].

Massive Open Online Courses (MOOCs) are open to a worldwide audience of participants via the internet. They have been a popular remote learning method since 2012 [10]. MOOCs make use of traditional methods (reading materials, videos of lectures, or online exams), but also provide interactive components (users’ forums and discussions). The latter foster engagement, facilitating interactions between participants, facilitators, and experts.

MOOCs could be a tool to decrease the training gap between research in evidence-based psychotherapy, and its implementation in the field. However, some limitations to the format of this course and its content could interfere with its implementation. To illustrate the potential and the challenges of this online format, we will take into account a survey that was performed during the First European Psychiatric Association (EPA) MOOC. This MOOC on CBT took place between April and May 2018.

## MOOCs Increase Dissemination and Accessibility of the Training

MOOCs are available online, and a weekly cycle of work is suggested to the students, so that they can do the training at their own pace. This also allows synchronization of the topic being revised by the students, fostering interaction on online forums. A facilitation team can help the students to reflect on their prior knowledge concerning the topic, and how to implement their new knowledge to answer their weekly quiz.

**Figure 1.**
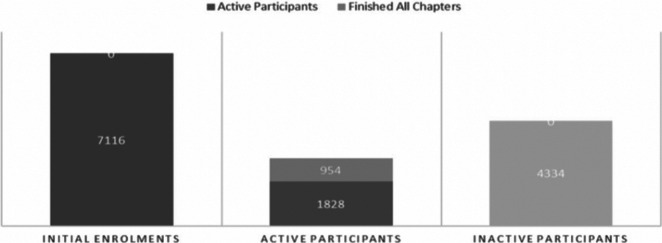
Enrolments and activity of the participants of the 1st EPA MOOC about CBT

For the first EPA MOOC, initially 7,116 participants enrolled, with 1,828 (26%) completing the quiz at the end of the first week (Figure 1). The cost-free model of the MOOC allowed for easy promotion of the course on social media, mailing lists and trainee networks.

Since the active time frame can be limited for some, the EPA MOOC allowed an extended access to the contents of the MOOC for 6 months after the 4 weeks of recommended completion time. Furthermore, several sessions can be organized with the same video and quiz materials.

Since the internet has no borders, it naturally allows for a large dissemination. For the first EPA MOOC, the majority of participants (76%) were European. The five most represented countries in screen watch time (accounting for 44% of the total) were Greece (18%), France (8%), United Kingdom (7%), Poland (6%), and Russia (5%). The remaining 56% came from 44 other countries.

Accessibility is so increased that it can allow the involvement of nonprofessionals as well. In the EPA MOOC, psychologists and psychology trainees were the most represented professional group (41%), followed by psychiatrists and psychiatry trainees (32%), but other professions included mental health practitioners, nurses, caregivers, patients, social workers, teachers, students from other areas, and diverse medical specialists. It could also be a tool of empowerment for patients and caregivers.

The technological aspect of a MOOC does not appear to be a limitation. The EPA MOOC was, for most of the participants (84%), their first MOOC, and a majority had never trained in CBT before (63%). About half of the participants (53%) had no previous experience in any other kind of psychotherapy.

As mentioned by Dias [5], subtitles could be necessary to teach on a global perspective for non-native English speakers.

The content of this MOOC was mostly introductory to the topic of CBT, but other online courses can be longer and can go into more detail, for example, the Beck Institute Course [4], the Pter Course [11]. The educational outcomes, even if uncontrolled, seem good. The average score of 21.4 out of 25 on the first week raised continuously every week up to 23.13 out of 25 in the fourth, and last, week.

## But the Engagement Needs to be Improved

In many MOOCs engagement is a big issue. There is a large mismatch between the initial enrolments and the active participants by the end. In the first EPA MOOC, only 954 (13%) of the initial 7,116 enrolments completed the course; still, this is about twice the average retention rate of MOOCs [9]. The topic and the quality of the contents may explain the relatively high retention rate, as suggested by participant feedback. The quality of the instructors had a pivotal role in registering for and attending this course (82%). The topic played a “very important” (80%) and “important” (19%) role for participants. We also hypothesize that this could have been due to the large underlying demand for training in psychotherapy [1]. This MOOC perhaps associated the ease of access to participants who already had basic knowledge on psychology or psychiatry.

Still, engagement of the participants could be improved. Patient simulation supported by avatar patients has been used in motivational interviewing during face-to-face training, and could be relevant during MOOCs for higher engagement and retention [12,13]. More interesting forms of peer assessment could replace traditional quizzes, and have been tried with success in other MOOCs. With the consent of patients, analysis of recordings of real sessions could also be used and deconstructed into their components, and alternative to this would be recordings of scripted sessions with actors.

## A Hybrid Approach Seems Relevant with the MOOC as an Incentive

Success in completing a MOOC does not represent clinical competency in delivering CBT. Even if MOOCs can be more interactive and involving than a book, they cannot replace face-to-face CBT training. They also cannot replace role-play exercises, and certainly not supervised clinical experience. Accreditation of clinical competence remains challenging with fully online models. We believe that hybrid systems could be relevant; e-learning could be used for the delivery of the fundamental theoretical topics, followed by an optional face-to-face module, including supervised exams and assessments of clinical competency. Making the optional face-to-face module subject to a payment seems reasonable in this context. A starting point for this could be the model used for the Human Brain project summer schools [14]. The EPA organized a summer school on psychotherapy for 4 years. About 25 trainees attended yearly, coming from the whole of Europe. During the last session in 2019, the participants were asked to follow the MOOC as a prerequisite of the summer school. A prior completion of the MOOC was required for the participants to allow them to gain basic theoretical knowledge beforehand, and thus to focus on more practical aspects of the training during the summer school.

We think that this initial training could be motivational in engaging in a more classical training, for example, with the Members’ Association of the European Association of Behavioral and Cognitive Therapies.

## Beyond the Promotion of the Topic, a MOOC Can be a Promotion Tool for the Provider

Before this MOOC, 3% of the participants knew about the EPA, but only 5% were members. A MOOC can be a means to improve visibility of a scientific association, to improve members, or congress participation (e.g., French MOOCs improved the visibility of universities by 2%). This increase was equivalent to climbing 75 ranks in the Shanghai ranking [15]. Since the cost does not change with the scale (it is not all that different if the materials are used by just one or several thousands of participants), a joint effort between different European Training Associations for promotion and funding could be fruitful.

## The Economic Model of the MOOC Needed to be Taken into Account

The economic model of a MOOC is challenging. MOOCs, by definition, provide free access, but some of them offer certification only to paying participants, to limit costs for the provider. That allows for a wide and open access, but payment is a requirement for the most engaged participants, who wish to certify their training. Free access fosters accessibility and dissemination of the course. During the promotion of the second EPA online course, some online communities were less likely to participate in a course that was not free anymore. They did not want to be involved in a commercial event, even if the fees to register were not sufficient to cover the development of the course.

From the survey carried out during the first MOOC, the fact that the course that was free was significant for 89% of the participants (“very important” to 45% and “important” to 44%).

## Conclusion

Online learning offers new possibilities to begin to fill the training gap in psychotherapy reported by psychiatry and psychology students. This MOOC of the EPA was an encouraging trial, with a large audience and good feedback. A sustainable economic model needs to be developed to allow for future developments.
